# Chronic fatigue and headache in post-COVID-19 syndrome: a radiological and clinical evaluation

**DOI:** 10.3389/fneur.2024.1526130

**Published:** 2025-01-22

**Authors:** Gonçalo G. Almeida, Saide Alkan, Robert Hoepner, André Euler, Lara Diem, Franca Wagner

**Affiliations:** ^1^Department of Radiology, Kantonsspital Baden, Baden, Switzerland; ^2^Affiliated Hospital for Research and Teaching of the Faculty of Medicine of the University of Zurich, Zurich, Switzerland; ^3^Switzerland Center for Neuroradiology, Klinik Hirslanden, Zurich, Switzerland; ^4^University Institute of Diagnostic and Interventional Neuroradiology, Inselspital, Bern University Hospital, University of Bern, Bern, Switzerland; ^5^Department of Neurology, Inselspital, Bern University Hospital, University of Bern, Bern, Switzerland; ^6^Department of Neurology, Cantonal Hospital Lucerne, Lucerne, Switzerland

**Keywords:** post-COVID-19 syndrome, MRI, white matter lesions, brain, spine, fatigue, headache, sleep disturbance

## Abstract

**Introduction:**

The coronavirus disease 2019 (COVID-19) pandemic has caused millions of infections and deaths globally. Post-COVID-19 syndrome, or long COVID is characterized by lingering symptoms such as chronic fatigue, headaches, and sleep disturbances. This study aimed to investigate the correlation between these symptoms and T2-hyperintense white matter lesions detected on magnetic resonance imaging (MRI) of the brain and spine in patients with post-COVID-19 syndrome.

**Methods:**

This retrospective, single-center study analyzed a sample of 96 patients from Bern University Hospital in Switzerland who presented with suspected post-COVID-19 syndrome between 2020 and 2022. Patients completed self-report questionnaires evaluating fatigue, emotional wellbeing, and daytime sleepiness. Brain and spine MRIs were independently rated by 2 neuroradiologists for T2-hyperintense lesions. The correlation between these lesions and symptoms of fatigue and headache was assessed.

**Results:**

The cohort consisted predominantly of women (73%) with an average age of 46 years. Chronic fatigue (90%), sleep disorders (51%), and headache (57%) were the most prevalent symptoms. The fatigue questionnaires indicated high levels of fatigue. Brain MRI revealed T2-hyperintense lesions in 72% of patients, whereas spine MRI showed these lesions in only 16%. There was no statistically significant correlation between the presence of cerebral T2-hyperintense lesions and symptoms of fatigue (*p* = 0.815) or headaches (*p* = 0.178). Similarly, no significant correlation was found when considering numbers of pathological brain lesions (fatigue: *p* = 0.557; headaches: *p* = 0.820).

**Conclusion:**

While T2-hyperintense lesions are common in patients with post-COVID-19 syndrome, their presence does not correlate significantly with symptoms of fatigue or headaches. These findings suggest that T2-hyperintense brain lesions may not be directly related to the subjective experience of these symptoms. Further research with larger sample sizes and adjustment for potential confounding factors is necessary to better understand the relationship between MRI findings and post-COVID-19 syndrome symptoms.

## 1 Introduction

The coronavirus disease 2019 (SARS-CoV-2) is a viral strain first reported in China in 2019, which rapidly evolved into a global pandemic responsible for more than 700 million infections and more than 7 million deaths reported worldwide as of April 2024 (1, 2). Infection-associated symptoms are multisystemic, possibly affecting the respiratory, cardiovascular, gastrointestinal, musculoskeletal, and neurologic systems. Most infected patients exhibit mild symptoms not requiring hospitalization (3). Although symptoms fully resolve in most cases, in over 10% of cases patients will experience lingering complications of infection, such as chronic fatigue and headaches, months after the initial infection (4–6). The finding of post-COVID-19 syndrome has been defined as “persistent, new, or recurrent symptoms and conditions more than 4 weeks after initial COVID-19 diagnosis” (7, 8).

Neurologic effects of COVID-19 on the central nervous system (CNS) have been reported since the start of the pandemic (9) and may be related to various mechanisms such as underlying systemic disease, immune dysfunction, vasculopathy, and complications of prolonged illness or hospitalization (6, 10, 11). The most common sequelae include chronic fatigue, headaches, sleep disorders, and depression (12, 13). A number of questionnaires have been validated for the clinical evaluation of these conditions, including the Fatigue Severity Scale (FSS), the Fatigue Scale for Motor and Cognitive Functions (FSMC) (14, 15), the Epworth Sleepiness Scale (ESS) (16, 17) and the Beck Depression Inventory (BDI-II) (18, 19). Some of these have also been applied to patients diagnosed with post-COVID-19 syndrome (20).

Overall, post-COVID-19 syndrome is associated with more than 60 heterogeneous physical and psychological symptoms affecting multiple organ systems (21). This heterogeneity has led to controversial and confusing findings in medical imaging research. In a large longitudinal brain imaging study, significant effects of SARS-CoV-2 infection, including a reduction in gray matter thickness and global brain size were reported (22). In contrast, Yiping et al. reported higher bilateral gray matter volume with no significant change in white matter volume in COVID-positive patients (23). Imaging findings tend to be nonspecific and are often seen as T2-hyperintense white matter lesions or supratentorial susceptibility abnormalities suggestive of microvascular pathology (24, 25). T2-hyperintense lesions are associated with small vessel disease, inflammatory processes and post-infectious sequelae, which may play a role in the pathophysiology of post-COVID-19 syndrome (26, 27). Despite conflicting reports in the literature, such as differences in lesion prevalence and significance in symptomatic patients (28), these lesions remain a widely studied marker of neurological abnormalities in post-viral syndromes.

The McDonald diagnostic criteria are commonly used in clinical practice as a way to standardize and provide diagnostic accuracy in the identification of multiple sclerosis (MS) (29). The revised McDonald criteria (2024) are expected to place more emphasis on early diagnosis. This will be achieved through advanced imaging techniques such as susceptibility-weighted imaging (SWI) and diffusion-weighted imaging (DWI), as well as certain imaging and laboratory biomarkers, such as the central vein sign (CVS) and paramagnetic rim lesions, among others (30, 31). The CVS represents the imaging manifestation of the perivenular nature of demyelinating plaques and has been defined as a hypointensity appearing at the center of a surrounding hyperintense lesion in at least 2 of 3 orthogonal planes. Although not pathognomonic for MS, the CVS can help differentiate between MS and other demyelinating diseases of the CNS (32, 33).

The exact relationship between these imaging findings and different post-COVID-19 syndrome symptoms remains unclear. The aim of this study was therefore to compare the number of T2-hyperintense white matter lesions in the brain and within the spinal cord among patients with post-COVID-19 syndrome exhibiting either chronic fatigue and/or headaches after initial infection.

## 2 Materials and methods

### 2.1 Ethics

We retrospectively evaluated clinical and paraclinical data on patients with post-COVID-19 syndrome included in the neuroimmunological registry (registration no. KEK-BE 2017–01369), treated at the neuroimmunological outpatient department of the Inselspital, University Hospital Bern, a tertiary care hospital. We analyzed the medical records of all patients with post-COVID-19 syndrome who had given informed consent. Only patients with MRIs performed in our neuroradiology department were included to ensure comparability of the images and availability of the necessary MRI sequences.

### 2.2 Materials

This retrospective, single-center study analyzed a sample of 96 patients from Bern University Hospital in Switzerland, who presented following COVID-19 infection between November 2020 and May 2022. All patients were confirmed to have had acute COVID-19 infection and persistent symptoms consistent with post-COVID-19 syndrome and presented at the neurology department for post-COVID-19 consultation.

### 2.3 Methods

Patients were selected based on their presentation to the Post-COVID-19 clinic with neurological symptoms such as fatigue and headache. Inclusion criteria required MRI imaging of the brain and/or spine and completion of validated symptom questionnaires. Imaging was performed on a 3T MRI scanner using standardized protocols, including for all patients an axial diffusion-weighted imaging sequences (DWI) with a slice thickness (ST) of 4 mm, an axial T2-weighted image sequence (ST 4 mm), a native T1-weighted MPR (ST 1 mm) and an axial susceptibility weighted imaging (SWI) sequence (ST 1.2 mm). After contrast application a 3D FLAIR sequence (ST 1 mm), an axial T1-weighted TSE (ST 4 mm) and a 3D T1-weighted MPR were acquired. If the patient presented with visual disturbances a native coronal T2-weighted fad suppressed sequence covering the orbits was added and after contrast application additional coronal T1- and T2-weighted sequences with fad suppression over the orbits were acquired. The standard spine protocol included the following sequences, covering the whole spine: coronal STIR, native sagittal T1- and T2-weighted imaging sequences and post contrast sagittal T1- and PD-weighted imaging sequences. In case of a pathological finding dedicated axial T2-weighted sequences and post contrast axial T1-weighted images were added. Table 1 shows the imaging protocols.

**Table 1:** Brain and spine MRI protocols.

Spine MRI		Brain MRI	
Native sequence	sag T2 sag T1 cor STIR	Native sequence	ax DWI ax T2 + PD sag 3D T1 MPRAGE ax SWI cor T2 fs Orbita
Contrast medium (mmol/kg)	0.1	Contrast medium (mmol/kg)	0.1
Contrast medium sequences	sag PD ax T2 sag T1 ax T1	Contrast medium sequences	sag 3D T1 MPRAGE ax T1 TSE sag 3D FLAIR cor T2 fs Orbita cor T1 KM fs Orbita

MRI, magnetic resonance imaging; sag, sagittal; ax, axial; cor, coronal; fs, fat suppression.

During the first consultation, a clinical history was taken detailing the reported symptoms during the acute phase of COVID-19 infection, as well as the current symptoms. Patients completed questionnaires on fatigue (Fatigue Severity Scale [FSS]), the impact of fatigue on motor and cognitive function (Fatigue Scale for Motor and Cognitive Functions [FSMC]), emotional wellbeing (Beck Depression Inventory II [BDI-II]), and daytime sleepiness (Epworth Sleepiness Scale [ESS]). Laboratory parameters such as C-reactive protein and ferritin were also analyzed to exclude possible secondary causes for fatigue.

Brain and spine MRIs performed in the setting of post-COVID-19 syndrome were independently rated by one board-certified radiologists with 5 years’ experience and by one board certified neuroradiologist with over 15 years’ experience, for the presence, number, and location of T2-hyperintense lesions in the brain and the spinal cord. The incidence of lesions with CVS and paramagnetic rim was also assessed. Both readers were blinded to clinical history and patients symptoms including questionnaire results to reduce observer bias. Discrepancies in lesion counts between the two raters were resolved through a consensus reading.

### 2.4 Statistical analysis

Data are presented as mean with 95% confidence interval (95% CI) and comparative statistics (Mann–Whitney U test (MWU) and chi-squared test, respectively) were used. A *p*-value of 0.05 was assumed to be statistically significant.

### 2.5 Data sharing statement

In compliance with an open data approach, anonymized data of the cohort are available on request from the corresponding author.

## 3 Results

### 3.1 Cohort

The average age of the study population was 46.0 years (95% confidence interval (CI): 42.8–49.1) and 73% were female (70/96). The patients had their first consultation a mean of 35.8 days after the onset of acute infection (95% CI: 31.4–40.2). Ninety-one tested positive with the PCR/antigen test (95%) and only 2 tested positive for antibodies (2%). For 3 patients no information about the testing method was available. The sample was further categorized according to the associated comorbidities. Asthma was the most prevalent (8 out of 96 patients; 8%), followed by 7 reports of depression (7%). The data are summarized in Table 2.

**Table 2:** Patient characteristics at first consultation.

Age, years, mean (95% CI), *n*	46.0 (42.8–49.1), 96
Female, *n* (%)	70/96 (73%)
Time between onset of acute infection and first consultation, weeks, mean (95% CI), *n*	35.8 (31.4–40.2), 96
Positive PCR/antigen test, *n* (%)	91/96 (95%)
Positive antibody test, *n* (%)	2/96 (2%)
Comorbidities	
Arterial hypertonia, *n* (%)	4/96 (4%)
Metabolic syndrome, *n* (%)	2/96 (2%)
Sleep apnea syndrome, *n* (%)	1/96 (1%)
Depression, *n* (%)	7/96 (7%)
Rheumatological disorders, *n* (%)	2/96 (2%)
Multiple sclerosis, *n* (%)	4/96 (4%)
Hashimoto thyroiditis, *n* (%)	2/96 (2%)
Asthma, *n* (%)	8/96 (8%)
Neurodermatitis, *n* (%)	2/96 (2%)

### 3.2 COVID-19 infection symptoms—acute phase

Most of the patients reported fever (*n* = 57, 59%), followed by headache (*n* = 55, 57%), anosmia (*n* = 55, 57%), fatigue (*n* = 54, 56%), and cough (*n* = 52, 54%). Intubation was the rarest consequence of COVID-19 infection observed in this sample, reported in only one case (1%). All reported symptoms are listed in Table 3.

**Table 3:** Symptoms of acute COVID-19, *n* (%).

Headache, *n* (%)	55/96 (57%)
Fever, *n* (%)	57/96 (59%)
Anosmia, *n* (%)	55/96 (57%)
Dyspnea, n (%)	34/96 (35%)
Cough, *n* (%)	52/96 (54%)
Cold, *n* (%)	43/96 (45%)
Pain, *n* (%)	47/96 (49%)
Gastrointestinal symptoms, *n* (%)	17/96 (18%)
Fatigue, *n* (%)	54/96 (56%)
Sleep disturbance, *n* (%)	25/96 (26%)
Hospitalization, *n* (%)	15/96 (16%)
Intubation, *n* (%)	1/96 (1%)

### 3.3 Post-COVID-19 symptoms—first consultation

The first follow-up consultation post-COVID-19 infection aimed at the assessment of lingering symptoms, analysis of various laboratory parameters, and completion of self-report questionnaires. Fatigue was to the most reported symptom during the acute phase of COVID-19 infection and still affected the majority of patients (*n* = 86, 90%) at the first post-COVID appointment. Fifty-five patients reported recurrent headaches (57%) and 49 sleep disorders (51%).

Of the 82 patients assessed for depressive symptoms following COVID-19 infection, 23 (28%) reported depression. When asked about daytime sleepiness, 29 patients (36%) reported feeling sleepy during the day. A full list of the prevailing symptoms post-COVID-19 infection is provided in Table 4.

**Table 4:** Post-COVID-19 symptoms at first consultation.

Fatigue, *n* (%)		86/96 (90%)
Sleep disorders, *n* (%)		49/96 (51%)
Headache, *n* (%)		55/96 (57%)
Pain, *n* (%)		39/96 (40%)
Paresthesia, *n* (%)		17/96 (18%)
Dyspnea, *n* (%)		32/96 (33%)
Anosmia/Ageusia, *n* (%)		28/96 (29%)
Cough, *n* (%)		3/96 (3%)
Dizziness, *n* (%)		31/96 (32%)
Autonomic dysfunction, *n* (%)		18/96 (19%)
Dermatological symptoms, *n* (%)		8/86 (9%)
Gastroenterological symptoms, *n* (%)		11/96 (11%)
Tinnitus, *n* (%)		5/96 (5%)
Visual symptoms, *n* (%)		3/96 (3%)
	Depression, *n* (%)	23/82 (28%)
	Daytime sleepiness, *n* (%)	29/80 (36%)

### 3.4 Self-report questionnaire scores

The FSS was completed by 81 patients of the original patient sample of 96 to assess self-reported fatigue severity in daily activities. The mean score was 5.2 for a cut-off defined at 4.0 (95% CI: 66.2–74.9). The impact of these fatigue levels on daily performance was measured using the FSMC, with 79 patients averaging 70.5 (43.0 cut-off; 95% CI: 66.2–74.9).

Eighty patients completed the ESS, scoring a mean average of 9.2 (10.0 cut-off; 95% CI: 66.2–74.9). The BDI-II was filled in by 82 patients who scored an average of 16.6 (14.0 cut-off; 95% CI: 14.7–18.5). The results are given in Table 5.

**Table 5:** Scores of self-reported questionnaires at first consultation.

FSS, mean (95% CI), *n*	5.2 (4.9–5.5), 81
FSMC total, mean (95% CI), *n*	70.5 (66.2–74.9), 79
ESS, mean (95% CI)	9.2 (8.0–10.3), 80
BDI-II, mean (95% CI)	16.6 (14.7–18.5), 82

FSS, Fatigue Severity Scale; FSMC, Fatigue Scale for Motor and Cognitive Functions; ESS the Epworth Sleepiness Scale, BDI-II Beck Depression Inventory.

### 3.5 Brain MRI

Of the 96 patients, 88 underwent a brain MRI after COVID-19 diagnosis. A total of 5 patients had a known demyelinating disease such as MS and were excluded from further analysis. The time between COVID-19 infection and brain imaging averaged 37.8 weeks (95% CI: 32.5–43.1) for the remaining 83 patients. Although post-COVID-19 syndrome shows a clear female predominance, we found no sex-specific differences in MRI findings.

**Figure 1:**
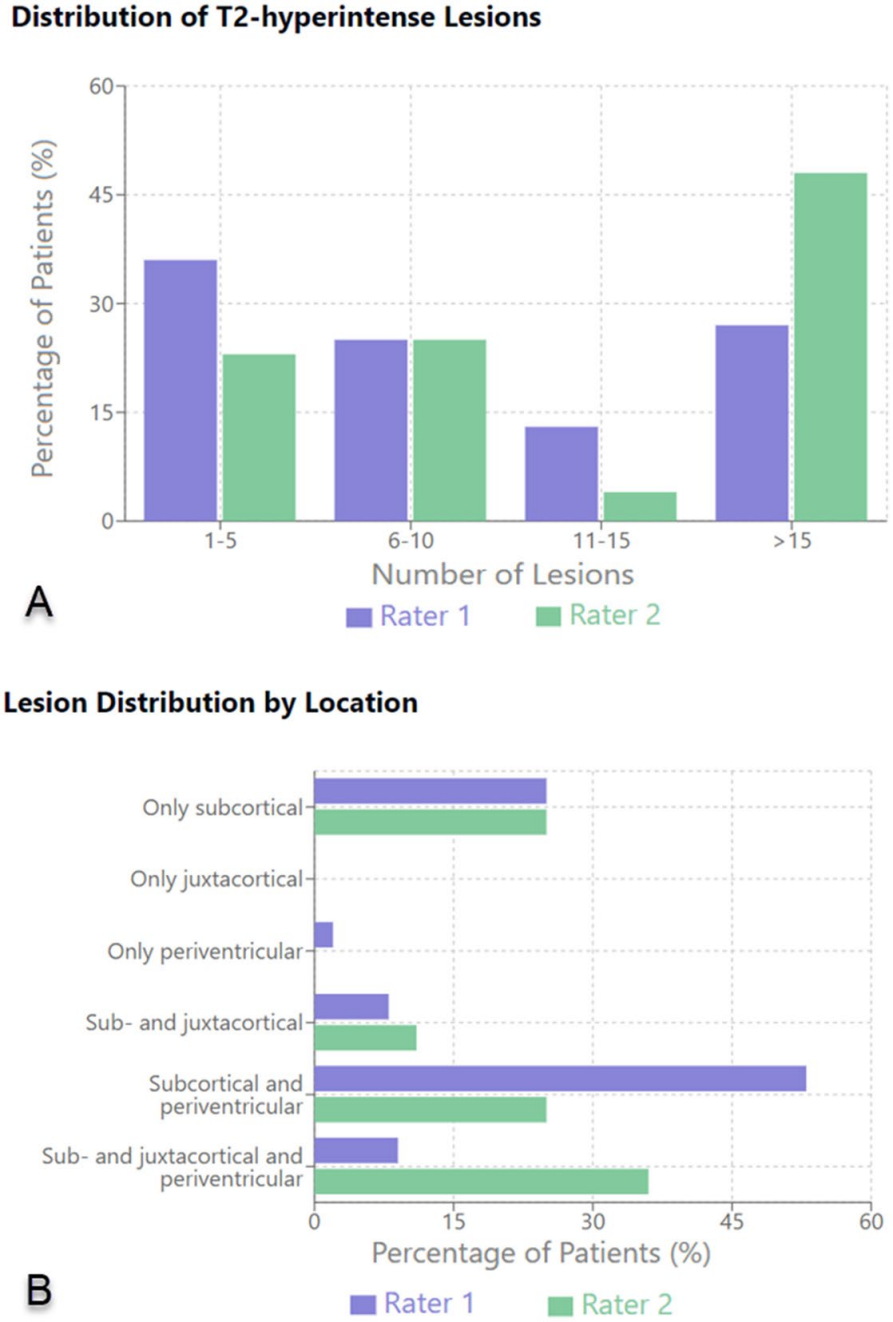
Distribution of T2-hyperintense lesions **(A)** and lesion distribution by location **(B)** on brain MRI.

The subsequent analysis of the MRIs was conducted independently by 2 radiologists. The first rater identified 64 patients with T2-hyperintense lesions, whereas the second identified 56. Both examiners then grouped the findings according to the total number and location of T2-hyperintense lesions as well as the number of lesions displaying a CVS. According to Rater 1 most patients (36%) had between 1 and 5 hyperintense lesions; 17 patients (27%) had more than 15 lesions; 16 (25%) had between 6 and 10 lesions and 8 patients (13%) had 11–15 T2-hyperintense lesions. Rater 2 reported that most patients (27; 48%) had more than 15 lesions, whereas only 4% had between 11 and 15 T2-hyperintense lesions. Neither of the raters found enhancing lesions or lesions with a paramagnetic rim. In the majority of cases no CVS was reported by either of the radiologists (Rater 1–75%; Rater 2–63%) with a decrease in incidence of patients displaying a higher number of lesions with CVS. As for the location of T2-hyperintense lesions, the first rater identified most lesions in the subcortical and periventricular areas (34 patients—53%), as well as 16 patients (25%) with only subcortical lesions. Rater 2 reported more patients showing sub- and juxtacortical lesions (20 patients—36%) and fewer with subcortical and periventricular lesions (14 patients—25%). Fifteen patients were reported by the second rater as having only subcortical T2-hyperintense lesions (25%). The results are shown in Table 6 and summarized in Figure 1.

**Table 6:** Findings of brain MRIs conducted in patients post-COVID-19 infection.

Cerebral MRI		
Number of patients with brain MRI after SARS-CoV-2 infection, *n* (%)	88/96 (92%)	
Number of patients with brain MRI and without demyelinating CNS disorders (e.g., multiple sclerosis) *n* (%)	83/96 (86%)	
Time between COVID-19 and brain MRI, weeks, mean (95% CI)	37.8 (32.5–43.1)	
Patients with T2-hyperintense lesions, *n* (%)	Rater 1	Rater 2
	64/83 (77%)	56/83 (67%)
Number of T2-hyperintense lesions, *n* (%)		
1–5	23/64 (36%)	13/56 (23%)
6–10	16/64 (25%)	14/56 (25%)
11–15	8/64 (13%)	2/56 (4%)
>15	17/64 (27%)	27/56 (48%)
Enhancing lesions	0/64 (0%)	0/56 (0%)
Lesions with paramagnetic rim	0/64 (0%)	0/56 (0%)
Lesions with CVS, *n* (%)		
None	48/64 (75%)	35/56 (63%)
1–2	10/64 (16%)	12/56 (21%)
3–4	6/64 (9%)	7/56 (13%)
5–6	0/64 (0%)	2/56 (4%)
T2-hyperintense lesions by location, *n* (%)		
Only subcortical	16/64 (25%)	14/56 (25%)
Only juxtacortical	0/64 (0%)	0/56 (0%)
Only periventricular	1/64 (2%)	0/56 (0%)
Only infratentorial	0/64 (0%)	0/56 (0%)
Sub- and juxtacortical	5/64 (8%)	6/56 (11%)
Subcortical and periventricular	34/64 (53%)	14/56 (25%)
Sub- and juxtacortical and periventricular	6/64 (9%)	20/56 (36%)
Subcortical, periventricular, infratentorial	2/64 (3%)	0/56 (0%)
All locations	0/64 (0%)	2/56 (4%)
Black holes	0/83 (0%)	0/83 (0%)
Leptomeningeal enhancement	1/79 (1%)	1/79 (1%)
Diffusion restriction	0/81 (0%)	0/81(0%)
Orbital/optic nerve pathologies	0/81 (0%)	0/81 (0%)
Only subcortical	16/64 (25%)	14/56 (25%)

MRI, magnetic resonance imaging; CNS, central nervous system; CVS central vein sign.

### 3.6 Spine MRI

A collection of spine MRIs was analyzed in a similar way to the brain MRIs. Of 95 patients, 19 had undergone spine MRI following infection with SARS-CoV-2. For 19 of 95 patients, no demyelinating CNS disorders were identified. The average time elapsed between COVID-19 infection and spine imaging was 36.1 weeks (95% CI: 19.9–52.3).

The same 2 raters conducted the evaluation of the spine MRIs. Both raters recorded a similar number of lesions across all spine regions with one third of patients showing between 1 and 3 lesions, most of which (67%) were in the cervical spine. All enhancing spine lesions appeared at the thoracic level. Table 7 summarizes the findings.

**Table 7:** Findings of spine MRIs conducted in patients post-COVID-19 infection.

Spine MRI		
Number of patients with spine MRI after SARS-CoV-2 infection, *n* (%)	19/95 (20%)	
Number of patients with spine MRI and without demyelinating CNS disorders (e.g., multiple sclerosis), *n* (%)	19/90 (20%)	
Time between COVID-19 and spine MRI, weeks, mean (95% CI)	36.1 (19.9–52.3)	
T2-hyperintense lesions, *n* (%)	Rater 1	Rater 2
	3/19 (16%)	3/19 (16%)
Number of T2-hyperintense lesions, *n* (%)		
1	1/3 (33%)	1/3 (33%)
2	1/3 (33%)	1/3 (33%)
3	1/3 (33%)	1/3 (33%)
T2-hypertintense lesions by location, *n* (%)		
Cervical spine	2/3 (67%)	2/3 (67%)
Thoracic spine	1/3 (33%)	1/3 (33%)
Enhanced lesions (thoracic spine)	1/3 (33%)	1/3 (33%)

MRI, magnetic resonance imaging; CNS, central nervous system.

## 4 Correlation between symptoms and brain MRI

This study considered the 2 most reported symptoms persisting after SARS-CoV-2 infection—headache and fatigue—and tried to determine the relationship between the prevalence of these symptoms and the number and location of T2-hyperintense lesions identified on brain MRI in this cohort of patients. The statistical analysis is depicted in Table 8.

**Table 8:** Statistical analysis comparing the number of T2-lesions in the brain between patients reported as having headaches or who were assessed as having chronic fatigue after COVID-19 infection.

	Patients without fatigue (FSS < 4.0)	Patients with fatigue (FSS > 4.0)	*p*-value
Number of T2-lesions in brain MRI, mean (95% CI)	6.7 (3.1–10.4), 14	6.4 (4.9–7.9), 59	0.815
	Patients without headache	Patients with headache	
Number of T2-lesions in brain MRI, mean (95% CI)	7.4 (5.3–9.6), 31	5.9 (4.3–7.5), 53	0.178
	Patients without fatigue (FSS < 4.0)	Patients with fatigue (FSS > 4.0)	*p*-value
Patients with pathological number of T2 lesions for age, *n* (%)	5/14 (35%)	27/59 (46%)	0.557
	Patients without headache	Patients with headache	
Patients with pathological number of T2 lesions for age, *n* (%)	13/31 (42%)	24/53 (45%)	0.820

FSS, Fatigue Severity Scale.

Due to the small number of patients with T2-hyperintense lesions identified on spine MRI, a similar statistical analysis was not possible for these data.

### 4.1 Headaches in patients with post-COVID-19 syndrome

For the 41 patients presenting with no headache, 31 had T2-lesions identified on brain MRI with a mean value of 7.4 (95% CI: 5.3–9.6), whereas for the 55 subjects presenting with headaches, the mean value was 5.9 (95% CI: 4.3–7.5, *p*-value 0.557). Of the 31 patients presenting with headaches and having T2-lesions identified on brain MRI, 13 showed a pathological number of T2-lesions according to age. In the “no headache” group of 31 individuals with T2-lesions, 24 were observed to have a pathological number of lesions for their age group (*p*-value 0.820).

### 4.2 Fatigue in patients with post-COVID-19 syndrome

For the 14 patients presenting with no fatigue (FSS < 4.0), a mean of 6.7 (95% CI: 3.1–10.4) T2-lesions were identified in brain MRI. The subjects identified as having fatigue (FSS ≥ 4.0) had a mean of 6.4 T2-lesions (95% CI: 4.9–7.9, *p*-value 0.815). Of the 14 individuals with no fatigue, 5 were diagnosed with a pathological number of T2-lesions for their age whereas, of the 59 patients with fatigue, 27 had a pathological number of lesions for their age (p-value 0.557).

## 5 Discussion

This study focused on radiological evaluation with brain/spine MRI in participants with post-COVID-19 syndrome in single-center cohort in Switzerland. In our cohort, the majority of patients suffering from Post-COVID-19 Syndrome were female (73%; 70/96). This gender predominance aligns with findings from previous studies on long COVID, which have shown that women are disproportionately affected by persistent symptoms (34). Several factors may contribute to this gender disparity: biological differences, such as sex-specific variations in immune response are well-documented with women tending to have stronger immune responses making them more prone to immune-mediated conditions, which could influence susceptibility to long COVID (35). Additionally, hormonal differences, particularly the modulatory effects of estrogen on immune and vascular function, may play a role in the manifestation of chronic post-viral symptoms (35). Beyond biological factors, gender-specific reporting and healthcare-seeking behaviors may further contribute to the observed differences, as women may be more likely to report symptoms and seek medical attention for chronic conditions like Post-COVID-19 Syndrome (36). These considerations underscore the need to account for gender differences when interpreting findings and developing management strategies for long COVID.

Our study underscores the complexity of interpreting radiological findings in patients with post-COVID-19 syndrome. This is particularly evident with respect to structural brain alterations seen on MRI and their relationship with the most commonly identified symptoms such as chronic fatigue, headaches and depression. MRI is often used to identify possible structural causes for persistent neurological symptoms. The most common finding in brain MRI in our study was the presence of T2-hyperintense lesions in subcortical and periventricular areas, which is in line with the current literature (37). These lesions have previously been reported in patients suffering from persistent fatigue after hospitalization for COVID-19 (26), as well as in those with post-COVID-19 “brain fog” (28). However, these lesions tend to be frequently encountered in other unrelated settings such as post-infectious and inflammatory conditions, as well as in cases of chronic hypertension and small vessel disease. Advanced MRI techniques, such as diffusion tensor imaging and functional MRI (fMRI) have been shown to help in identifying imaging alterations that seem to be more prevalent in patients exhibiting neurological symptoms in the setting of post-COVID-19 syndrome (23, 38). However, our study did not find a significant correlation between reported radiological abnormalities and the most common symptoms reported by our patient cohort.

Symptoms such as chronic fatigue could be influenced by a range of physiological and psychological factors, including immune dysregulation, hormonal imbalances, and mental health conditions like depression or anxiety (21). The findings of our study suggest that these complex factors may not manifest as detectable structural changes in the brain, at least not in ways that are visible using conventional MRI protocols. While more complex imaging protocols such as fMRI or advanced diffusion techniques may be more sensitive to subtle brain changes, in our experience their application in clinical practice remains limited. Other options, such as detailed clinical assessments and the use of validated questionnaires for symptom tracking, may be a more effective strategy in guiding therapy for patients presenting with post-COVID-19 syndrome (39), than using MRI as a routine follow-up tool. This could shift the focus of treatment strategies from purely neurological investigations to a more multidisciplinary approach including neuropsychiatric, psychological, and rehabilitative care.

Transverse myelitis, while uncommon, has been documented in the literature as a severe complication of viral infections, including COVID-19 (27). In our study, only 3 patients demonstrated spine lesions consistent with post-infectious myelitis. This low prevalence aligns with current findings that transverse myelitis is a rare but serious post-COVID-19 complication, typically linked to more severe neurological symptoms such as motor weakness and sensory deficits (27). This suggests reserving spine MRI for cases where more specific neurological deficits are present, rather than incorporating it as part of the routine post-COVID-19 workup.

Our findings suggest a need to re-evaluate the role of MRI in managing patients with post-COVID-19 syndrome. The absence of a clear structural correlation with common symptoms such as fatigue and headaches indicates that MRI findings, while useful in excluding structural brain abnormalities, may not provide actionable insights for guiding therapeutic interventions. As such, the nonspecific nature of white matter lesions calls for a cautious approach when attributing patient symptoms to these findings. Instead, functional and neuropsychological assessments, including cognitive testing and fatigue scales, might yield more direct information about the patient’s condition and better inform therapeutic strategies.

Our study has some limitations. Firstly, the sample size is relatively small, particularly that for the analysis of spine MRI data, which limits the statistical power of the findings. Secondly, this study is retrospective and single-center, which may limit the generalizability of the results. The subjective nature of self-report questionnaires could introduce bias in the evaluation of symptoms. Additionally, the study did not adjust for other potential confounding factors such as treatment received during acute COVID-19 infection. Furthermore, this study relies on a single time-point for imaging and symptom assessment. Whilst previous studies suggest that some imaging abnormalities, such as those associated with inflammation or microvascular injury, may resolve or change with symptom recovery (38, 39), additional longitudinal studies with repeated imaging at multiple timepoints—such as 1, 3, and 6 months post-infection—are needed to evaluate the progression and resolution of T2-hyperintense lesions over time and to validate our findings.

While T2-hyperintense lesions are commonly observed in patients with post-COVID-19 syndrome, their presence does not seem to significantly correlate with symptoms of fatigue or headaches. These findings suggest that T2-hyperintense lesions may not be directly related to the subjective experience of these symptoms in patients with post-COVID-19 syndrome. Further research with larger sample sizes and adjustment for potential confounding factors is necessary to better understand the relationship between MRI findings and post-COVID-19 syndrome.

## Data Availability

The raw data supporting the conclusions of this article will be made available by the authors, without undue reservation. Requests to access the datasets included in the neuroimmunological registry (registration no. KEK-BE 2017-01369) and treated at the neuroimmunological outpatient department of the Inselspital, University Hospital Bern, should be directed to franca.wagner@insel.ch.
